# Job Burnout, Mood State, and Cardiovascular Variable Changes of Doctors and Nurses in a Children's Hospital in China

**DOI:** 10.1155/2014/386719

**Published:** 2014-03-09

**Authors:** Shuchang He, Yan Chen, Junya Zhan, Johnna Wu, Mark Opler

**Affiliations:** ^1^Department of Psychology, Peking University, No. 5 Yiheyuan Road, Haidian District, Beijing 100871, China; ^2^Capital Institute of Pediatrics, Beijing 100020, China; ^3^Department of Psychiatry, New York University School of Medicine, New York City, NY 10016, USA

## Abstract

*Aims*. This study examines mood and cardiovascular variables related to job stress and burnout in hospital personnel. *Main Methods*. 400 nurses and physicians from a children's hospital in China were recruited. Participants completed job stress, burnout, and mood state questionnaires. Cardiovascular variables such as body mass index (BMI), triglyceride (TG), and high density lipoprotein (HDL) were measured. *Key Findings*. Job stress and burnout were significantly associated with mood state. Statistically significant correlations were found between triglyceride levels and job stress scores (*r* = 0.175, *P* < 0.01), BMI and job stress scores (*r* = 0.121, *P* < 0.05), and HDL levels and job stress scores (*r* = −0.117, *P* < 0.05). *Significance*. Mood state changes may be related to job stress and job burnout, in turn, associated with triglycerides and HDL levels. Public health implications and interventions are discussed.

## 1. Introduction

Burnout is a psychosomatic syndrome characterized by the three core dimensions of emotional exhaustion (EE), feelings of depersonalization (DP), and reduced personal accomplishment (PA) along with mental weariness. Its etiology is generally thought to be long-standing stress at work or elsewhere [1]. Burnout is an important occupational problem in doctors and nurses [2] since they have continuous and constant contact with patient morbidities and mortalities [3]. Burnout affects nearly one in two salaried physicians and pharmacists in France [4]. A survey in a German university hospital found that more than half of the physicians/psychologists, a quarter of the nursing staff, and 10% of the remaining employees suffered from work-related mental stress disorders. Physicians and psychologists were especially affected by psychosomatic symptoms [5]. A three-hospital survey in China showed that surgeons and physicians had the highest scores of job burnout [6].

Among the three dimensions, emotion exhaustion is regarded as the principal dimension associated with the correlates and consequence of burnout [7]. It is important to study mood states because they not only influence the individuals in their work condition but they also have a work-family interface and are related to their behavior in their families as well [8]. Negative emotions are regarded as a plausible dispositional factor responsible for job satisfaction and influence the way people perceive their working environments [9]. The people with more positive mood states are more optimistic, are more likely to give higher quality services, and are more likely to have a greater sense of accomplishment, while negative moods are associated with giving poor service and increased absenteeism and turnover [10, 11]. More frequent negative moods are expected to contribute to tendencies to depersonalize patients [12].

Many published studies focus on emotional experiences at work [13]; they are not only associated with the performance and attitude of the individuals at work but are also related to their cardiovascular system activation [14]. Stress can influence the cardiovascular system via activated hypothalamic-pituitary-adrenocortical (HPA) and sympathoadrenomedullary (SAM) axes. Many documents demonstrate the possible association of job stress and cardiovascular heart disease [15]. High strain and active jobs are related to increased cardiovascular risk among women [16]. In a recent review on the autonomic nervous system and cardiovascular risk factors, Thayer and colleagues found that decreased vagal function is a risk factor for all-cause mortality and that work stress is associated with decreased HRV [17].

## 2. Methods

*Participant.* This study was conducted in Beijing, including doctors (132) and nurses (184) from a children's hospital. 400 questionnaires were distributed and a total of 316 questionnaires were completed and collected (the response rate was 79%). This study was approved by the Human Subject Ethics Committee of Department of Psychology at Peking University. The ethics committees of the hospital also approved the study. The demographic variable characteristic of the participants is listed in Table 1.

## 3. Questionnaire

The questionnaires included four parts. Part one was a demographic and general condition information section (e.g., age, gender, education year, department, marriage, etc.). General medical variables for each participant such as body avoirdupois, stature, blood pressure, blood lipid level, and blood glucose levels were obtained from their physical examinations within the last 12 months.

Part two was a job stress questionnaire, developed by Wang Lei and collaborators (Wang et al.). Wang's instrument includes 16 items scored on a Likert scale, ranging from 1 (never) to 7 (always); total scores reflect overall job stress. Job stress was divided into four levels according to the Lei scale total score: relaxed (<40), normal (40–64), overload (65–75), and much overload (>75).

Part three was comprised of the Profile of Mood States (POMS) from the Neurobehavioral Core Test Battery (NCTB) recommended by the World Health Organization, including 65 questions focusing on six domains; anger-hostility (POMSA), confusion-bewilderment (POMSC), depression-dejection (POMSD), fatigue-inertia (POMSF), tension-anxiety (POMST), and vigor-activity (POMSV). Participants responded to each item on a 5-point scale, ranging from 1 (never) to 5 (always) using the past week as a reference.

Part four involved assessment of job burnout. A Chinese version of the 15-item Maslach Burnout Inventory (MBI) [18] was utilized. This instrument includes three subscales that measured emotional exhaustion (EE), depersonalization (DP), and lack of personal accomplishment (PA). Responses to each MBI item ranged from never (0) to every day (6). Subscale scores were computed as the sum of the responses made to each item in a given subscale. Burnout was defined by high scores on the EE and DP subscales and low scores on the PA subscale [18]. A relative absence of burnout was characterized by the opposite pattern.

## 4. Statistics 

Student's *t*-test, variance analysis, and Chi-square test were used to make comparisons. Pearson correlations were computed to examine the association among scores in three main dimensions, job stress, mood state scores, and cardiovascular variables.

## 5. Results 

### 5.1. Job Stress

The total scores of job stress in the 316 participants were from 21 to 103.

The mean value was 60.81 ± 16.31 (see Table 2).

### 5.2. Job Burnout

Our data showed that the mean scores on the job burnout subscales were 22.04 ± 8.34 for emotional exhaustion (EE), 13.47 ± 6.52 for feelings of depersonalization (DP), and 15.72 ± 6.40 for reduced personal accomplishment (PA) (see Table 3). These values indicated a moderate to higher level of emotion exhaustion, a moderate level of depersonalization, and a much higher level of personal accomplishments in the nurses and doctors.

*T*-test showed that the scores of the three dimensions of job burnout had no significant difference between male and female participants (see Table 3).

Comparing the scores of the three dimensions regarding the age, sex, marriage, years of education, and the department, results showed that no significant difference was observed between men and women with regard to the scores of three dimensions of job burnout. The participants >40 years old showed slightly higher scores on the EE scale. Participants 30–39 years old showed higher scores on the DP and PA scales. Married individuals had higher scores on EE and DP compared with unmarried ones. Participants from different departments also showed significantly different EE scores among medicine, surgery, and others (see Table 3).

### 5.3. Mood State

No significant differences were observed when comparing scores of mood state between men and women. However, there were significant differences among age groups with regard to the six mood factors. There were also significant differences by years of education in mood state scores. Comparison between married participants and unmarried ones showed that there were significant differences with regard to the six mood factors (*P* < 0.001). Comparing the mood scores of participants in different departments, we observed that the scores of POMST, POMSA, POMSF, and POMSC significantly differed between different departments (see Table 4).

Correlational analyses demonstrated that job stress scores were statistically significant and positively related to scores of emotion exhaustion, depersonalization, and reduced personal accomplishment (*P* < 0.01) (see Table 5).

A statistically significant positive correlation emerged between scores of the three dimensions of job burnout and scores of negative mood state (POMSA, POMSC, POMSD, POMSF, and POMST), and negative correlations were seen between scores of the three dimensions of job burnout and POMSV scores (*P* < 0.01) (see Table 6). Statistically significant positive correlations were found between blood triglyceride level and job stress scores (*r* = 0.175, *P* < 0.01) and BMI and job stress (*r* = 0.121, *P* < 0.05). A statistically significant negative correlation was found between blood HDL level and job stress scores (*r* = −0.117, *P* < 0.05). No statistically significant correlations were found between blood glucose and job stress, diastolic pressure and job stress, and systolic pressure and job stress (see Table 7).

## 6. Discussion

This study examined associations between job stress, job burnout, mood state, and cardiovascular variables in doctors and nurses from a children's hospital in Beijing. Results show that approximately 18% of the participants experience significant job stress. Job stress and associated job burnout was in turn related to mood changes. Increasing job stress may be related to higher negative moods and reduced positive moods. Participants with higher scores of job stress and job burnout may require further evaluation, considering that negative mood not only influenced health but also influenced job performance.

There were no differences between men and women in mood scores on the three dimensions of job burnout. A study from Sweden found differences in working conditions and social networks between women and men with burnout [19]. Patients with burnout differ from the general population with respect to individual and social factors as well.

One of the more intriguing findings from our study was the positive correlation between job stress scores and serum triglyceride levels. The negative correlation between job stress and HDL levels suggests that job stress might increase triglycerides and decrease HDL. Prior studies in Beijing have demonstrated changes in coronary heart disease and cholesterol levels over time [20]. High blood HDL level is regarded as a protective factor for the cardiovascular system. Consequently, the decreased blood HDL levels related to job stress may be a useful risk marker for patients in occupational settings. Prior findings suggest that job strain may be a modifiable risk factor for metabolic syndrome and subsequent cardiovascular disease [21]. A survey in China by Xu et al. found that high levels of effort, overcommitment, and low reward increased the risk of dyslipidemia among Chinese workers, and they were significantly associated with triglycerides and LDL rather than HDL or other factors. Changes in blood lipids may be the possible link between job stress and coronary heart disease [22], although prior findings are equivocal [23].

The negative correlations between BMI and job stress scores indicate that BMI may also be a potentially useful biomarker. A previous study showed that increases in BMI (1 kg/m^2^) raised the risk for diabetes mellitus by approximately 26% in nonobese male Japanese [24]. Increased BMI may be a possible risk factor for diabetes in workplaces. A study found that increased BMI may occur because workers feeling stressed are prone to change their eating behavior and eat too much, which results in obesity [25].

Our study builds on the prior lines of evidence for the role of job strain as burnout syndrome seems to be a real independent cardiovascular risk factor. Training to manage emotions may increase cardiac vagal tone and may be cardioprotective [26]. Learning to control mood states and increasing positive mood may be an important intervention strategy to reduce job stress related to cardiovascular health.

## 7. Conclusion

This study concludes that job stress is related to three dimensions of job burnout, mood states, and cardiovascular variables in the doctors and nurses in a hospital setting in Beijing. High job stress and negative mood states appear to be associated with increased risk of developing cardiovascular diseases. We conclude that reduction of job stress is critical for prevention of cardiovascular diseases in the work place in order to foster a higher quality of life.

## Figures and Tables

**Table 1:** The general condition of participants.

Item	Number	Percentage
Male	38	12.0
Female	278	88.0
Physician	132	41.8
Nurse	184	58.2
No position	82	25.9
Primary	124	39.2
Middle	77	24.4
Pre-high	23	7.3
High	10	3.2
High school and/or below	128	40.5
University	107	33.9
Graduate school	81	25.6
Medicine	129	40.8
Surgery	87	27.5
Emergency	52	16.5
Others	48	15.2
Unmarried	100	31.6
Married	213	67.4
Divorced	2	0.6
Widowed	1	0.3

**Table 2:** Job stress scores of participants.

Item	Relax	Normal	Much stress	Overload
Number	39	145	75	57
Percentage	12.3	45.9	23.7	18.0

**Table 3:** Job burnout scores of different demographic variables.

Demographic characteristics	*n *	EE	DP	PA
Sex				
Male	38	4.2 ± 1.5	3.2 ± 1.5	2.5 ± 1.0
Female	278	4.4 ± 1.7	3.4 ± 1.7	2.6 ± 1.1
*t *		−0.781	−0.69	−1.015
*P *		0.435	0.491	0.311
Age				
20–29	152	4.1 ± 0.1	3.1 ± 0.1	2.7 ± 0.1
30–39	108	4.6 ± 0.2	3.7 ± 0.2	2.8 ± 0.1
≥40	56	4.8 ± 0.2	3.4 ± 0.2	2.2 ± 0.1
*F *		5.622	4.049	5.706
*P *		0.004	0.018	0.004
Education				
High school or below	128	4.5 ± 0.1	3.4 ± 0.1	2.6 ± 0.1
University	107	4.6 ± 0.2	3.5 ± 0.2	2.7 ± 0.1
Graduate school	81	4.0 ± 0.2	3.0 ± 0.2	2.5 ± 0.1
*F *		3.867	2.254	1.102
*P *		0.022	0.107	0.334
Department				
Medicine	129	4.6 ± 0.1	3.3 ± 0.1	2.6 ± 0.1
Surgery	87	4.5 ± 0.2	3.6 ± 0.2	2.6 ± 0.1
Emergency	52	4.6 ± 0.3	3.5 ± 0.2	2.9 ± 0.1
Others	48	3.7 ± 0.2	2.9 ± 0.2	2.3 ± 0.2
*F *		3.348	2.057	2.607
*P *		0.02	0.11	0.05
Is married^A^				
Unmarried	100	3.8 ± 1.7	2.8 ± 1.6	2.6 ± 1.1
Married	213	4.7 ± 1.6	3.6 ± 1.6	2.7 ± 1.1
*t *		−4.224	−4.497	−0.664
*P *		<0.001	<0.001	0.507

^A^There are also two divorced and one widowed.

**Table 4:** POMS scores of different demographic variables.

Demographic characteristic	*n *	POMST	POMSD	POMSA	POMSV	POMSF	POMSC
Sex							
Male	38	11.7 ± 7.6	16.1 ± 14.6	14.7 ± 11.0	17.0 ± 5.9	9.9 ± 7.0	9.7 ± 5.3
Female	278	12.1 ± 6.5	16.7 ± 11.9	15.0 ± 9.9	15.7 ± 6.1	10.8 ± 6.1	9.8 ± 4.5
*t *		−0.349	−0.267	−0.188	1.187	−0.864	−0.136
*P *		0.727	0.789	0.851	0.236	0.388	0.892
Age							
20–29	152	11.0 ± 0.5	14.0 ± 1.0	13.3 ± 0.8	17.3 ± 0.5	9.2 ± 0.5	9.0 ± 0.4
30–39	108	13.0 ± 0.6	19.0 ± 1.2	16.7 ± 1.0	14.1 ± 0.6	12.0 ± 0.6	10.8 ± 0.4
≥40	56	12.7 ± 0.9	19.2 ± 1.6	16.1 ± 1.3	15.5 ± 0.8	12.2 ± 0.8	10.4 ± 0.6
*F *		3.304	7.068	4.068	9.362	8.871	5.342
*P *		0.038	0.001	0.018	<0.001	<0.001	0.005
Education							
High school or below	128	11.5 ± 0.6	15.3 ± 1.1	14.5 ± 0.9	16.6 ± 0.5	10.4 ± 0.6	9.3 ± 0.4
University	107	12.5 ± 0.6	18.9 ± 1.2	16.6 ± 1.0	14.7 ± 0.6	11.7 ± 0.6	10.6 ± 0.4
Graduate school	81	12.2 ± 0.7	15.7 ± 1.4	13.6 ± 1.1	16.3 ± 0.7	9.8 ± 0.7	9.6 ± 0.5
*F *		0.746	2.843	2.414	3.182	2.288	2.272
*P *		0.475	0.06	0.091	0.043	0.103	0.105
Department							
Medicine	129	12.7 ± 0.6	17.4 ± 1.1	15.2 ± 0.9	15.7 ± 0.5	11.3 ± 0.5	10.2 ± 0.4
Surgery	87	11.3 ± 0.7	16.2 ± 1.3	15.4 ± 1.1	16.4 ± 0.7	10.4 ± 0.7	9.9 ± 0.5
Emergency	52	13.4 ± 0.9	18.2 ± 1.7	17.3 ± 1.4	15.2 ± 0.8	11.8 ± 0.9	10.2 ± 0.6
Others	48	10.1 ± 1.0	13.5 ± 1.8	11.0 ± 1.4	16.4 ± 0.9	8.4 ± 0.9	8.1 ± 0.7
*F *		2.924	1.533	3.519	0.571	3.216	2.646
*P *		0.034	0.206	0.015	0.634	0.023	0.049
Is married^A^							
Unmarried	100	10.2 ± 6.3	12.2 ± 10.8	11.8 ± 9.9	18.2 ± 5.4	8.2 ± 5.8	8.4 ± 4.4
Married	213	12.9 ± 6.6	18.6 ± 12.3	16.4 ± 9.7	14.8 ± 6.1	11.8 ± 6.1	10.5 ± 4.5
*t *		−3.459	−4.48	−3.931	4.861	−4.934	−3.713
*P *		0.001	<0.001	<0.001	<0.001	<0.001	<0.001

^A^There are also two divorced and one widowed.

**Table 5:** Coefficient of correlation (*r*) of job stress and job burnout (*n* = 316).

	Job stress	EE	DP	PA
Job stress	1			
EE	0.562**	1		
DP	0.474**	0.699**	1	
RP	0.287**	0.68**	0.358**	1

**P < 0.01.

**Table 6:** Coefficient of correlation (*r*) of job burnout and mood states (*n* = 316).

	POMST	POMSD	POMSA	POMSV	POMSF	POMSC
EE	0.516**	0.551**	0.480**	−0.425**	0.603**	0.481**
DP	0.503**	0.550**	0.510**	−0.314**	0.521**	0.486**
PA	0.331**	0.351**	0.278**	−0.283**	0.320**	0.305**

**P < 0.01.

**Table 7:** Coefficient of correlation (*r*) of job stress and cardiovascular variables (*n* = 316).

	Blood glucose	Diastolic pressure	Systolic pressure	TG	HDL	BMI	Job stress
Blood glucose	1						
Diastolic pressure	0.126*	1					
Systolic pressure	0.037	0.239**	1				
TG	0.332**	0.448**	0.099	1			
HDL	−0.458**	−0.328**	−0.076	−0.716**	1		
BMI	0.167**	0.474**	0.220**	0.403**	−0.332**	1	
Job stress	−0.002	0.081	0.061	0.175**	−0.117*	0.121*	1

HDL: high density lipoprotein. TG: triglyceride. *P < 0.05; **P < 0.01.
